# Prospective Assessment of Intraoperative Facial Nerve Monitoring in Patients Undergoing Partial Parotidectomy

**DOI:** 10.1155/2022/3318175

**Published:** 2022-03-22

**Authors:** Maciej Zieliński, Paweł Sowa, Monika Adamczyk-Sowa, Michał Szlęzak, Maciej Misiołek

**Affiliations:** ^1^Department of Otorhinolaryngology and Oncological Laryngology in Zabrze, Faculty of Medical Sciences in Zabrze, Medical University of Silesia, Katowice, Poland; ^2^Department of Neurology in Zabrze, Faculty of Medical Sciences in Zabrze, Medical University of Silesia, Katowice, Poland; ^3^Fizjosport Medical Center, Gliwice, Poland

## Abstract

The most significant complication of parotid gland tumor surgery is facial weakness. This study compares the occurrence of transient facial palsy in patients with parotid gland tumors who underwent surgery without monitoring to those who underwent surgery with monitoring. The study's aim was to investigate facial nerve function in patients undergoing parotidectomy as well as the effect of intraoperative facial nerve monitoring and the effect of certain risk factors on the surgery and onset of postoperative facial palsy. This prospective study included 100 patients who underwent parotidectomy. The study cohort was divided into two groups. Group I included 50 patients who underwent surgery without neuromonitoring and group II included 50 patients who underwent surgery with neuromonitoring. The neurological assessment was conducted using the House–Brackmann scale. Preoperatively and one month postoperatively, electroneuronography (ENoG) and blink reflex tests were done. The analyses showed a significant reduction of the compound muscle action potential (CMAP) amplitude of the orbicularis oculi and orbicularis oris muscles and prolonged R1 and R2 blink reflex latencies 1 month after surgery. On neurological and electrophysiological studies, the rate of postoperative transient facial nerve dysfunction was significantly different between the groups. Significantly more patients, operated with use of facial nerve monitoring, presented postoperatively normal nerve function (i.e., House–Brackmann grade I) compared to those who underwent surgery without monitoring (78% and 26%, respectively; *p* < 0.001). Monitoring had a statistically significant impact on the prevalence of facial nerve conduction disorders in patients who underwent surgery, according to the blink reflex and ENoG studies. The duration of the surgical procedure was not affected by monitoring in any way. The clinical evaluation of facial nerve function (House–Brackmann scale) and some ENoG results 1 month after surgery were found to have a significant correlation. To summarize, using monitoring considerably reduced the negative impact of local factors and the prevalence of transient facial nerve palsy.

## 1. Background

Salivary gland tumor surgery is always associated with the risk of facial nerve injury due to the course of the facial nerve through the parotid gland parenchyma. This can result in postoperative palsy or paresis with impaired mobility of the mimic muscles on the affected side. According to the literature, permanent facial nerve paralysis occurs in 0%–7% of patients who undergo surgery for parotid gland tumors. Transient changes in facial nerve conduction affect up to 65% of patients [1–3].

Many factors influence the risk of paresis, including tumor type and location, histological type, extent and duration of the surgery, reoperation, intraoperative bleeding, and the need to dissect nerve branches from the tumor [3–5]. Surgical experience is also an important factor in the surgical outcome [6].

Minimizing the risk of permanent dysfunction of the facial nerve is a significant clinical problem. Intraoperative nerve function monitoring and advanced neuromonitoring devices have been introduced to support surgeons during the procedure. Currently, intraoperative monitoring of the facial nerve is performed using electromyography based on the recording of the compound muscle action potential (CMAP) from facial muscles. The CMAP occurs in response to electrical stimulation of the facial nerve with a probe or (even accidental) mechanical irritation of the nerve during tumor dissection from the gland. This method is widely used by head and neck surgeons, allowing intraoperative identification of the extracranial portion of the facial nerve that runs through the parotid gland parenchyma and minimizing the risk of damage.

Despite the increasing use of intraoperative facial nerve monitoring and many studies, no conclusive results show a reduced prevalence of facial nerve palsy in patients undergoing surgery for benign parotid tumors with monitoring [1, 2, 7, 8]. Some data have indicated a reduction in the prevalence of transient palsy of the facial nerve [2, 3]. Conversely, other studies have demonstrated no statistically significant effect [1, 8].

This study was performed due to the ambiguity of the research results. The aim was to prospectively assess the effect of the intraoperative neuromonitoring system on the occurrence of facial nerve paresis in a homogeneous group of patients scheduled for parotid gland tumor surgery compared with patients who had no monitoring. The electrophysiological assessment enabled an objective comparison of the pre- and postoperative function of the nerve using conventional electroneurography (ENoG) and blink reflex examinations [9]. In addition, we analyzed the influence of local and surgery-related factors on the occurrence of facial nerve palsy.

## 2. Methods

### 2.1. Study Subjects

This prospective study included 100 patients who underwent surgery for parotid gland tumors in the Department of Otorhinolaryngology and Laryngological Oncology in Zabrze, Medical University of Silesia in Katowice, Poland. The cohort was divided into two groups:

- Group I: 50 patients who underwent surgery between 2015 and 2016 without intraoperative facial nerve monitoring (34 women [68%] and 16 men [32%], aged 55.8 ± 15.16)
- Group II: 50 patients who underwent surgery for parotid gland tumors between 2016 and 2019 with facial nerve monitoring (32 women [64%] and 18 men [36%] aged 54.26 ± 14.58)

The neuromonitoring equipment was purchased in 2016, and the ongoing prospective study, which evaluated factors affecting facial nerve palsy, was extended by a group undergoing surgery with monitoring. All subsequent parotid surgical procedures were performed using neuromonitoring. Therefore, randomization was not used in this study.

The inclusion criteria were a primary benign tumor of the parotid gland diagnosed by ultrasound and fine-needle aspiration biopsy and no previous surgery in this region.

The exclusion criteria were tumor recurrence or malignancy of the salivary gland, history of other salivary gland diseases (sialolithiasis and Sjögren's syndrome), previous treatment of head and neck region cancer, tumors penetrating from the salivary gland into the parapharyngeal space, central facial nerve palsy, systemic diseases that could affect nerve function (e.g., alcoholic neuropathy), pacemaker, refused consent, or withdrawal from the study.

All subjects gave written informed consent. Patient data were protected. Study approval was obtained from the Bioethics Committee of the Medical University of Silesia in Katowice (no. KNW/0022/KB1/5/I/17, May 30, 2017). The study was conducted according to the ethical principles for medical research involving human subjects of the Declaration of Helsinki.

### 2.2. Surgical Treatment and the Assessment of Facial Nerve Function

All surgical procedures were performed using surgical loupes (2.5x magnification). Medtronic NIM-Response 3.0 (4-channel device; Medtronic Xomed Inc.) was used for intraoperative facial nerve monitoring. Stimuli of 100 microseconds and 0.8 mA were used. The signaling threshold was at least 100 *μ*V.

Patients were not selected based on the tumor location in the parotid gland as it could result in the exclusion of more difficult cases, adversely affecting the study objectivity. This enabled the use of monitoring in patients with tumors in the deep lobe to be assessed. In such cases, we performed a partial parotidectomy, which included both the superficial lobe and the portion of the deep lobe of the parotid gland. The surgeon decided the extent of surgery. The primary goal was facial nerve preservation while sufficiently performing radical surgery.

### 2.3. Evaluation of the Facial Nerve Function

Each patient in the study group underwent pre- and postoperative assessment of facial nerve function using the House–Brackmann scale and electrophysiological evaluation. Before surgery, no patient had facial nerve paresis (based on the House–Brackmann scale). Facial nerve injury was classified as transient if the function returned within six months after the surgery. However, if the dysfunction lasted for more than six months, facial nerve injury was classified as permanent. The 6-month follow-up and electrophysiological tests that had been planned were canceled. After 6 months, all the patients had normal nerve function on clinical examination.

Electrophysiological studies of the facial nerve included the blink reflex test and bilateral assessment of the facial nerve using ENoG. The electrophysiological studies were performed in the Department of Neurology EMG Laboratory, using Neuro-Mep-4 (Neurosoft, Russia). Excitability, terminal latency, standardized latency, and the evoked potential amplitude were assessed.

The amplitude of the CMAP is affected by age, skin thickness, temperature, and environmental humidity. The EMG Laboratory's reference ranges were used. The change in absolute CMAP amplitude between the first and second examinations and the ratio of CMAP amplitudes between the symptomatic and asymptomatic sides were included in the study. The facial nerve was stimulated at the preauricular area during electroneurography, while surface receiving electrodes were placed on the skin above the orbicularis oculi and orbicularis oris muscles. A ground electrode was placed on the forehead. The nerve was irritated on both sides with a single 15-100 mA electrical stimulus; it was repeated several times every 5 seconds and increased until the maximal CMAP occurs (supramaximal stimulation).

The examination of the blink reflex involved the bilateral recording of the contraction of orbicularis oculi muscles in response to unilateral supraorbital electrical stimulation. Receiving electrodes were placed laterally and below the outer angles of the eyes while the reference electrodes on the lateral part of the nose and the grounding electrode on the forehead. A stimulating electrode was applied over the supraorbital foramen, at the exit of the supraorbital nerve. The supraorbital nerve was irritated sequentially on both sides with a single 15–20 mA electrical stimulus repeated at least six times. The latencies of the R1 and R2 waves of the blink reflex (expressed in ms) were assessed. The reference values of the latencies adopted in the study were <11.5 ms for R1 and <35 ms for R2.

Demographic data, clinical features of the tumor, the extent and course of surgery, and surgical observations were collected with a postoperative questionnaire, which included the following parameters: tumor size and location, histological type, type of surgery, the severity of bleeding, need for dissection of the facial nerve from the tumor (wrapping), monitoring problems, and surgical prediction of whether the continuity of the nerve was interrupted and whether facial nerve palsy might occur. Tumor size was categorized based on the largest tumor diameter (2 cm, 2–4 cm, and >4 cm). Tumor subsites were categorized into three compartments based on the surgical findings of the anatomical relationship to the facial nerve. These were superficial lobe, deep lobe, and both superficial and deep lobes, with the tumor crossing the facial nerve plane.

### 2.4. Statistical Analyses

Statistical analyses were performed using IBM SPSS Statistics (Windows, Version 25.0. Armonk, NY: IBM Corp). First, descriptive statistics were analyzed for the normality of distribution. Comparisons between the patient groups were conducted for categorical variables using the chi-square test of independence or Fisher's exact test. Analyses using Student's *t*-test for independent samples or the Mann–Whitney *U* test were conducted to compare the groups in terms of quantitative and ordinal variables. Correlation analysis to determine the variables' relationships was performed using Kendall's *τ* coefficient.

Next, ordinal regression and logistic regression were performed. *Α* = 0.05 was adopted as the significance level. A mixed-design analysis of variance was performed to test the differences between the first and second electrophysiological parameter measurements of the affected side considering the between-group factor (i.e., the presence of monitoring).

## 3. Results

The groups were similar in terms of demographics, tumor histological characteristics, and size. However, the tumor was significantly more often located in the deep lobe in group II (Table 1).

The mean surgery time in patients without monitoring was 100.4 min (SD = 32.95), while in the group with monitoring, it was 100.98 min (SD = 42.88). The times were not statistically significantly different.

### 3.1. Degree of Postoperative Facial Nerve Dysfunction

The analysis showed significant differences in the prevalence of facial nerve paresis between the groups (*p* < 0.001; *V* = 0.55). In 78% of patients (*n* = 39) in group II, there was no deterioration of the facial nerve function. This result was significantly higher than group I, which was 26% (*n* = 13). Group II had a significantly lower percentage of patients with discrete and moderate facial nerve paresis (House–Brackmann grades II and III) than group I. More severe dysfunction of the facial nerve (House–Brackmann grades IV–VI) was noted in group II, whereas in group I, grade IV paresis occurred in eight patients (16%) and grade V in two patients (4%). None of the patients presented with complete facial nerve paralysis. The results are given in Table 2.

Facial nerve branches were wrapped around the tumor in 48 (48%) cases (group I, 26, 52%; group II, 22, 44%). This was not significantly different between the groups (*p* = 0.423). In the patients with tumor wrapping, there was no facial nerve dysfunction in five patients (19.2%) in group I and 16 patients (72.7%) in group II, 1 month postoperatively.

Normal facial nerve function was recovered in all patients within six months (House–Brackmann grade I). This shows that the facial nerve's continuity was preserved in all cases. There were no signs of damage to the central or peripheral nervous system.

### 3.2. Facial Nerve (ENoG)

The CMAP amplitude from the orbicularis oculi muscle was assessed in the whole study group. The analysis showed significantly lower CMAP amplitude 1 month postoperatively (*M* = 0.38; SE = 0.03) compared to preoperative assessment (*M* = 0.60; SE = 0.04) (*p* < 0.001). The amplitude analysis showed significant differences between the first and second ENoG studies in group I (*p* < 0.001). In the second study, patients had a significantly lower amplitude from the orbicularis oculi muscle than the first study (decreased amplitude by 0.38, on average; SD = 0.47). A decrease in the amplitude was also noted in group II (decrease in the amplitude by 0.07, on average; SD = 0.31). However, this difference was not statistically significant (*p* = 0.197) (Table 3).

Next, the analysis of measurements of the CMAP amplitude from the orbicularis oris muscle was performed for the affected side pre- and postoperatively. A significant decrease in the CMAP amplitude was found postoperatively (*M* = 0.85; SE = 0.07) compared to the preoperative study (*M* = 1.19; SE = 0.09) in the whole group of patients (*p* = 0.003).

The decrease in the CMAP amplitude of the orbicularis oris muscle in both groups was similar and was not statistically different (*p* = 0.816) (Table 3).

### 3.3. Blink Reflex Test

The R1 and R2 latencies (ms) on the affected side were analyzed. The reference values of latencies adopted in the study were 11.5 ms for R1 and <35 ms for R2 and R2'.

The analysis showed significant differences in R1 latency between the first and second studies in group I (*p* < 0.001). In the postoperative study, patients had a significantly longer R1 latency than in the preoperative study. However, in group II, the difference between the measurements was not significant (*p* = 0.660).

In the whole group, the R2 latency (*M* = 37.47; SE = 1.26) was significantly longer in the postoperative study than the preoperative study (*M* = 34.38; SE = 1.12) (*p* = 0.043).

Patients in group I (*M* = 39.22; SE = 1.30) had a significantly longer R2 latency than patients in group II (*M* = 32.63; SE = 1.30). The difference between the groups and between the studies was not significant (*p* = 0.113, Table 4).

### 3.4. Relationship between Electrophysiological Findings and the Results of the Clinical Examination (House–Brackmann Scale)

Several correlation analyses with Kendall's *τ* were performed to determine the relationship between the electrophysiological findings and clinical examinations. The analyses included the clinical examination results (House–Brackmann scale) 1 month postoperatively, and the electrophysiological findings expressed as the ratio of the CMAP amplitude of the affected side to the unaffected side, the standardized latency, and the R1 and R2 latencies of the blink reflex.

The relationship between the electrophysiological and clinical findings 1 month postoperatively was significant for all parameters except R2 latency. Positive weak to moderate relationships were observed between the clinical examination results and the standardized latencies of the responses from the orbicularis oculi muscle, the orbicularis oris muscle, and R1 and R2 latencies. In other words, the higher the values of these parameters, the higher the scores on the House–Brackmann scale. Negative and weak relationships were observed between the clinical examination results and the amplitudes of the responses from the orbicularis oculi and the orbicularis oris muscles (the higher the amplitude, the lower the scores on the House–Brackmann scale). The ratio of the affected side to the unaffected side was used to compare the relationships of the ENoG results (CMAP amplitude and the standardized latency).

### 3.5. Analysis of Risk Factors for Postoperative Facial Nerve Paresis

Logistic regression analysis was performed to determine which of the analyzed clinical parameters were significant predictors of facial nerve paresis. We aimed to create a model to identify patients at a higher risk of postoperative facial nerve dysfunction.

With facial nerve paresis as the dependent variable, the patients were divided into two groups: with paresis (House–Brackmann grades II-V) and without paresis (House–Brackmann grade I).

The independent variables included selected clinical parameters and surgery-related variables (gender; histological tumor type: mixed tumor and Warthin's tumor vs. other types; tumor size: <2 cm vs. ≥2 cm; tumor location: superficial lobe, deep lobe, superficial, and deep lobes; intraoperative bleeding: large vs. small or medium; and the nerve wrapped around the tumor).

The model allowed the prediction of facial nerve paresis better than the model considering the constant only (*χ*^2^(8) = 4.27; *p* = 0.832). Nagelkerke's R2 of 0.415 indicated that 42% of the variance of the dependent variable could be predicted based on the model. The prediction success was 76% (75% in patients with paresis and 76.9% in the group without paresis), which suggested a moderately accurate prediction of paresis based on the analyzed model.

The analysis showed that intraoperative facial nerve monitoring was the only significant predictor of paresis. In monitored patients, the probability of paresis was reduced by 92% compared to the unmonitored patients. The influence of tumor size on the prediction of paresis was also observed. If the tumor was larger than 2 cm, the probability of paresis was increased 3.34-fold than patients with a smaller tumor (<2 cm). Other parameters were not significant predictors of paresis. Detailed results of the analysis are given in Table 5.

## 4. Discussion

Parotid gland surgical procedures vary due to the location, size, histological type of the tumor, and the course of the facial nerve branches. A large superficial tumor in the inferior portion of the superficial lobe is less surgically challenging than a small malignant tumor close to the facial nerve trunk. For this reason, it is extremely difficult to design objective studies that reliably assess the effectiveness of intraoperative facial nerve monitoring during parotid surgery. Malignant tumors can infiltrate branches of the facial nerve and require a radical treatment strategy. They cannot be compared with benign tumors or be used in evaluating the effectiveness of intraoperative facial nerve monitoring. Therefore, patients with malignant tumors were excluded from this study. Pleomorphic adenoma (51%) and Warthin's tumor (37%) were the most prevalent tumors seen. The prevalence of different histological tumor types in the study group is consistent with the epidemiological data from the literature [10].

In the current study, transient facial nerve palsy (House–Brackmann grade ≥ 2) occurred in 48% of patients, consistent with other studies [1, 11]. The results of studies that have evaluated the effect of intraoperative monitoring on the prevalence of facial nerve palsy after parotidectomy are inconclusive [1, 2, 8, 12]. Grosheva et al. found no statistically significant difference (*p* = 0.2) between the groups of patients who underwent surgery with and without monitoring in terms of the prevalence of facial nerve paresis [12]. The authors compared two groups of patients who underwent superficial parotidectomy for benign tumors of the parotid gland in this study. Facial nerve monitoring and visual observation of mimic muscle movements were used in 41 patients, while visual observation alone was used in 38 patients.

In a meta-analysis that included 546 patients, Sood et al. showed a statistically significantly lower prevalence of transient deterioration of facial nerve function in monitored patients (22.5% vs. 34.9%; *p* = 0.001). No significant difference was found between the groups in the occurrence of permanent paralysis (3.9% vs. 7.1%; *p* = 0.18) [2].

Significant efficacy of intraoperative facial nerve monitoring was also reported by Savvas et al. [13], who evaluated patients undergoing superficial parotidectomy. They observed normal facial nerve function in 85.4% of 123 patients who underwent surgery with monitoring and in 53.5% of 99 patients in the control group at the first postoperative evaluation. The results may have been influenced by the team's experience as the monitored group underwent surgery 12 years after the control group. The authors also reported no effect of monitoring on the duration of surgery [13].

In our study, a normal result (House–Brackmann grade I) was obtained by significantly more patients in group II than in group I, 1 month postoperatively (78% vs. 26%, *p* < 0.001). The analysis also showed significant electrophysiological differences between the groups in the prevalence of facial nerve dysfunction after parotidectomy.

Facial nerve monitoring allows for easier visualization of the facial nerve and its course, thus avoiding unwitting manipulation of its branches. Currently, surgeons tend to preserve as much parotid parenchyma as possible and perform extracapsular dissection (ECD) with adequate tissue margins whenever possible [5, 14–16].

In a retrospective study of 2988 patients who underwent ECD surgery with facial nerve monitoring, Bär et al. reported significantly decreased complications such as nerve palsy and the Frey syndrome compared to superficial parotidectomy [16]. Previously, Kadletz et al. obtained the opposite results in a retrospective study involving patients who underwent similar surgery, but without monitoring, at the University of Vienna [17]. The use of facial nerve monitoring by Bär et al. may have influenced the assessment of the efficacy and safety of ECD [16].

The most commonly reported risk factors for postoperative facial nerve paresis in the literature are older age, location in the deep lobe, size and histological malignancy of the tumor, the extent of parotid surgery, previous parotid surgery, facial nerve wrapped around the tumor, and longer duration of surgery [3–5, 18–20]. The significance of these factors is not entirely understood, as only some studies confirm their effect on facial nerve function [21]. Most studies evaluating complications after parotidectomy were retrospective and involved diverse groups of patients [22]. There are relatively few large prospective studies with a standardized follow-up and planned methodology for facial nerve assessment [1, 3, 5, 12]. The pathogenesis of postoperative facial nerve dysfunction is still the subject of many studies. Parotid tumor surgery may stretch the nerve and interfere with the nerve's vascularization. In such situations, there is a significant risk of neuropraxia caused by compression and ischemia. Coagulation near the nerve, the presence of necrotic tissue, and fibrosis are also important factors leading to postoperative facial nerve dysfunction [3–5, 12].

Our study results were different from the above studies. In our analysis, the lack of intraoperative monitoring was the only risk factor for facial nerve paresis. Based on the data obtained in this study, we calculated that the probability of paresis was reduced by 92% in patients with intraoperative monitoring during parotid surgery.

Assessment of the effect of tumor size (*p* = 0.06) showed that if the tumor was larger than 2 cm in diameter, the risk of paresis was increased 3.34-fold compared to patients with a tumor <2 cm. This could imply that it is more beneficial to remove benign parotid tumors early after diagnosis than to watch and wait until they reach >2 cm. Other investigated local risk factors did not prove to be significant. Patients with malignant tumors of the parotid gland were excluded from the study; however, neither pleomorphic adenoma nor Warthin's tumor increased the risk of paresis.

This analysis did not show a statistically significant difference in the duration of surgery between the study groups. However, the time of surgery was significantly longer in patients with tumors located in the deep lobe of the parotid gland or a portion of the lobes. A longer time of surgery was not a factor increasing the risk of facial nerve palsy. It is an additional parameter in the most difficult surgical procedures associated with a higher risk of facial nerve injury. Placing and securing electrodes and preparing the device for monitoring do not prolong the procedure duration. It is performed during surgical draping and takes approximately 5 minutes when performed by an experienced team.

Electrophysiological studies are an important part of the diagnosis of acute facial nerve injury [23]. These showed the influence of parotid surgery on the deterioration of facial nerve function in the whole study group 1 month postoperatively compared to the preoperative assessment. ENoG performed 1 month postoperatively showed decreased CMAP amplitude, both from the orbicularis oris and the orbicularis oculi muscles. However, the standardized latency did not change. In the blink reflex test, a statistically significant prolongation of R1 and R2 latencies was observed, which indicated that the integrity of the facial nerve fibers was often affected during parotid surgery. The monitored patient group significantly differed from the unmonitored group in some postoperative test results. Facial nerve monitoring significantly prevented a postoperative decrease in the CMAP amplitude from the orbicularis oculi muscle and prevented the prolongation of R1 latency in the blink reflex test. This suggests less damage to the axons and myelin sheaths of the upper branch of the facial nerve in group II patients.

There was no correlation between the electrophysiological findings and the clinical picture in the preoperative assessment. The ENoG and blink test parameters for individual nerve branches were abnormal in some patients, whereas none of the subjects presented with facial nerve paresis on clinical examination (House–Brackmann grade I). These results differ from those presented by Wiertel-Krawczuk et al., who found no electrophysiological or clinical abnormalities of the facial nerve in patients with benign parotid gland tumors on preoperative examination. However, they demonstrated a high correlation of ENoG results of the facial nerve and the blink reflex with the House–Brackmann score in follow-up examinations 1 and 6 months postoperatively [24].

In our study, the correlations between postoperative clinical findings (House–Brackmann scale) and electrophysiological findings 1 month after surgery (CMAP amplitude ratio of the affected side to the healthy side, standardized latency ratio of the affected side to the healthy side, and R1 latency of the blink reflex) were found to be statistically significant. Weak to moderate correlations may indicate clinical examination subjectivity and greater sensitivity of electrophysiological testing in diagnosing subclinical facial nerve injury. This would confirm Esslen's estimation that visible changes occur only when 50% of the nerve axons are damaged [25]. On the other hand, the House–Brackmann scale is designed to evaluate the entire facial nerve. Damage to a single branch affects the total assessment of the facial nerve function with this scale. ENoG, on the other hand, allows independent evaluation of the upper and lower extracranial portions of the facial nerve. The blink reflex test evaluates only the zygomatic branch of the facial nerve responsible for the orbicularis oculi muscle contraction. The specificity of the facial nerve damage during parotidectomy depends upon the degree of damage to individual nerve branches. Complete discontinuity of one branch can be reported with a simultaneous normal function of other branches. The House–Brackmann scale cannot fully describe the changes in nerve function in such cases. A more detailed scale would be more useful in ENT practice, e.g., the facial nerve grading scale 2.0 (FNGS 2.0) [26] or the postparotidectomy facial nerve grading system, which evaluates four nerve branches [27].

Monitoring seems to be most useful in difficult cases where the tumor is close to the nerve. Our study showed a significant difference between the groups when the facial nerve branches were wrapped around the tumor, and the tumor was located in the deep lobe.

No facial nerve dysfunction was observed (House–Brackmann grade I) 1 month postoperatively in 19.2% of monitored patients and 72.7% of monitored patients when nerve branches were wrapped around the tumor. The monitoring device is particularly useful in surgical procedures for malignant tumors of the parotid gland because it allows intraoperative confirmation of the extent of nerve damage when it is infiltrated by neoplastic cells.

In group II, the tumor occurred more frequently in the deep lobe of the parotid gland. In such cases, partial parotidectomy that also involves the deep lobe is required. Such a location creates a higher risk of facial nerve damage [3, 4]. Moreover, in patients in whom the tumor was located in the deep lobe of the parotid gland or both lobes, the median surgical time was 120 minutes, which was significantly higher than in patients whose tumor was in the superficial lobe (90 minutes). The use of intraoperative monitoring in patients with tumors in the deep lobe allowed the preservation of normal facial nerve function in 13 patients (68.4%) in group II compared to one patient (10%) in group I.

The surgeon confirmed the usefulness of the intraoperative facial nerve monitoring device in 47 cases (94%), as shown in the postoperative questionnaires. To adequately interpret the signal from the intraoperative facial nerve monitoring device, the surgeon must understand the technical and physiological principles underlying its use [28]. This maximizes its benefits and allows for correct management in the case of unexpected events. Otherwise, the device may become a distracting “gadget,” which could adversely affect the surgery.

We are well aware of the study's limitations. For more representative results, a larger study on randomized group would be required. We believe that our research will provide valuable information for future full-scale randomized trials.

## 5. Conclusions

In this study, the whole group showed a significant decrease in the CMAP amplitude from the orbicularis oris and the orbicularis oculi muscles (ENoG), and a statistically significant prolongation of R1 and R2 latencies was found 1 month postoperatively (blink reflex test). This shows that the blink reflex test and ENoG are sensitive methods for the objective assessment of facial nerve function in patients after parotid surgery. They could provide valuable support in ENT practice. Our study further demonstrated the safety of the intraoperative facial nerve monitoring device and its efficacy in reducing the prevalence of transient palsy of the facial nerve after surgery for benign parotid tumors. It does not affect surgery or its duration. The use of monitoring largely reduced the negative influence of local factors and significantly reduced the prevalence of transient facial nerve palsy. Therefore, the routine use of intraoperative facial nerve monitoring can be strongly recommended in all parotid surgical procedures.

## Figures and Tables

**Table 1:** Data of demographics, tumor features, and surgery length.

Parameters	All patients	Group I	Group II	*p*
Sex				
Women	66	34	32	0.673
Men	34	16	18	
Age (years; M) (SD)	54.96 (14.87)	55.80 (15.16)	54.26 (14.58)	0.606

Histological findings				
Pleomorphic adenoma	51	23	28	0.558
Warthin tumor	37	21	16	
Other	12	6	6	

Tumor size (maximal tumor diameter)				
<2 cm	24	10	14	0.206
2–4 cm	55	26	29	
>4 cm	21	14	7	

Location				
Superficial lobe	71	40	31	0.024
Deep lobe	21	5	16	
Superficial and deep lobes	8	5	3	

Time of surgery [min] (SD)	100.64 (38.05)	100.88 (42.88)	100.4 (32.95)	0.950

**Table 2:** Assessment of the facial nerve function in patients 1 month postoperatively.

Assessment based on the House–Brackman scale	*N*	%	Group I (*n* = 50)	Group II (*n* = 50)
			Total *n* (%)	Total *n* (%)
I	52	52	13 (26%)_a_	39 (78%)_b_
II	25	25	17 (34%)_a_	8 (16%)_b_
III	13	13	10 (20%)_a_	3 (6%)_b_
IV	8	8	8 (16%)_a_	0_b_
V	2	2	2 (4%)_a_	0_a_
VI	0	0	0_a_	0a

The values in the columns with different letter indices differ at the level of *p* < 0.05 (Bonferroni correction). Group I—patients who underwent surgery without facial nerve monitoring. Group II—patients who underwent surgery with facial nerve monitoring. *n*—group size.

**Table 3:** Summary of the results of amplitudes from the orbicularis oculi muscle and the orbicularis oris muscle.

	Examination	*M*	SE	95% CI	
				LL	UL
Orbicularis oculi muscle [mV]					
Group I	1	0.86	0.06	0.74	0.98
	2	0.48	0.05	0.38	0.58
Group II	1	0.35	0.06	0.23	0.47
	2	0.28	0.04	0.18	0.37

Orbicularis oris muscle [mV]					
Group I	1	1.19	0.13	0.93	1.45
	2	0.82	0.10	0.62	1.03
Group II	1	1.19	0.13	0.93	1.45
	2	0.88	0.10	0.68	1.08

Group I—patients undergoing surgery in whom facial nerve monitoring was not applied. Group II—patients undergoing surgery in whom facial nerve monitoring was applied. M: mean; SE: standard error; CI: confidence interval; LL and UL: lower and upper limits of the confidence interval.

**Table 4:** Summary of the results of R1 and R2 latencies of the blink reflex for the study groups.

	Study	*M*	SE	95% CI	
				LL	UL
R1 latency [ms]					
Group I	1	8.33	0.44	7.45	9.22
	2	11.43	0.43	10.58	12.28
Group II	1	8.87	0.44	7.99	9.75
	2	8.64	0.43	7.79	9.49

R2 latency [ms]					
Group I	1	36.46	1.58	33.33	39.59
	2	41.98	1.78	38.45	45.50
Group II	1	32.29	1.58	29.17	35.42
	2	32.97	1.78	29.44	36.50

Group I—patients undergoing surgery with facial nerve monitoring. Group II—patients undergoing surgery without facial nerve monitoring. M: mean; SE: standard error; CI: confidence interval; LL and UL: lower and upper limits of the confidence interval.

**Table 5:** Predictors of the occurrence of facial nerve paresis following salivary gland tumor surgery. Logistic regression coefficients.

Predictors	*B*	SE	*W*(1)	*p*	OR	95% *CI*	
						*LL*	*UL*
Facial nerve monitoring	−2.58	0.57	20.43	<0.001	0.08	0.03	0.23
Sex	−0.66	0.54	1.52	0.217	0.52	0.18	1.48
Tumor size	1.21	0.64	3.54	0.060	3.34	0.95	11.73
Tumor type	0.49	0.80	0.38	0.540	1.64	0.34	7.89
Tumor location			1.42	0.492			
Tumor location (1)	−0.54	1.08	0.25	0.616	0.58	0.07	4.86
Tumor location (2)	0.27	1.19	0.05	0.823	1.30	0.13	13.40
Bleeding	−0.09	0.82	0.01	0.910	0.91	0.18	4.53
Tumor wrapping	−0.42	0.52	0.63	0.426	0.66	0.24	1.84
Constant	2.06	1.47	1.96	0.162	7.85		

B: regression coefficient; SE: standard error; W: Wald test value; *p*: test probability; OR: odds ratio; CI: confidence interval; LL: lower limit; UL: upper limit; tumor location (1): superficial lobe; tumor location (2): deep lobe.

## Data Availability

The data that support the findings of this study are available on request from the corresponding author.
